# Association between personal exposure to ambient metals and respiratory disease in Italian adolescents: a cross-sectional study

**DOI:** 10.1186/s12890-016-0173-9

**Published:** 2016-01-12

**Authors:** Maria José Rosa, Chiara Benedetti, Marco Peli, Filippo Donna, Marco Nazzaro, Chiara Fedrighi, Silvia Zoni, Alessandro Marcon, Neil Zimmerman, Rosalind Wright, Roberto Lucchini

**Affiliations:** Department of Preventive Medicine, Icahn School of Medicine at Mount Sinai, One Gustave L. Levy Place, Box 1057, New York, NY 10029 USA; Department of Medical and Surgical Specialties, Radiological Sciences and Public Health, Section of Occupational Medicine, University of Brescia, Brescia, Italy; Department of Civil, Environmental, Architectural Engineering and Mathematics of the University of Brescia, Brescia, Italy; Department of Diagnostics and Public Health, Unit of Epidemiology & Medical Statistics, University of Verona, Verona, Italy; School of Health Sciences, Purdue University, West Lafayette, IN USA; Department of Pediatrics, Pulmonary and Critical Care, Icahn School of Medicine at ’Mount Sinai, New York, NY USA

**Keywords:** Metal, Personal monitoring, Adolescent health, Asthma, Air pollution

## Abstract

**Background:**

Release of ambient metals during ferroalloy production may be an important source of environmental exposure for nearby communities and exposure to these metals has been linked to adverse respiratory outcomes. We sought to characterize the association between personal air levels of metals and respiratory health in Italian adolescents living in communities with historic and current ferroalloy activity.

**Methods:**

As part of a study in the industrial province of Brescia, Italy, 410 adolescents aged 11–14 years were recruited. Participants were enrolled from three different communities with varying manganese (Mn) levels: Bagnolo Mella which has current ferroalloy activity, Valcamonica, which has historic ferroalloy activity and Garda Lake which has no history of ferroalloy activity. Particulate matter <10 μm in diameter (PM_10_) was collected for 24 h in filters using personal sampling. Mn, nickel (Ni), zinc (Zn), chromium (Cr) and iron (Fe) were measured in filters using x-ray fluorescence. Data on respiratory health was collected through questionnaire. Data for 280 adolescents were analyzed using a modified Poisson regression, and risk ratios were calculated for an interquartile (IQR) range increase in each pollutant.

**Results:**

In adjusted models including PM_10_ as a co-pollutant, we found significant associations between concentrations of Mn (RR: 1.09, 95 % CI [1.00, 1.18] per 42 ng/m^3^ increase), Ni (RR: 1.11, 95 % CI [1.03, 1.21] per 4 ng/m^3^ increase) and Cr (RR: 1.08, 95 % CI [1.06, 1.11] per 9 ng/m^3^ increase) and parental report of asthma. We also found significant associations between increased Mn and Ni and increased risk of asthma medication use in the past 12 months (RR: 1.13, 95 % CI [1.04, 1.29] and (RR: 1.13, 95 % CI [1.01, 1.27] respectively).

**Conclusions:**

Our findings suggest that exposure to ambient Mn, Ni and Cr may be associated with adverse respiratory outcomes.

**Electronic supplementary material:**

The online version of this article (doi:10.1186/s12890-016-0173-9) contains supplementary material, which is available to authorized users.

## Background

Ferroalloy production for steel manufacturing can release large amounts of metals, including manganese (Mn), nickel (Ni), zinc (Zn), chromium (Cr) and iron (Fe), into the atmosphere. The adverse respiratory effects of these fumes are well-documented in the occupational setting. A longitudinal study in Austria found that the duration of occupational exposure was significantly associated with decreases in lung function measures [1]. Other studies have reported increased respiratory symptoms and occupational asthma in workers exposed to welding fumes [2–4]. These emissions may be an important source of environmental exposure for populations in nearby residential communities. For example, a study of healthy subjects in Ontario, Canada found that lung function measures were significantly lower after subjects spent five consecutive days in a neighborhood adjacent to a steel plant when compared to 5 days spent on a college campus farther away [5]. Furthermore, even after the cessation of ferroalloy production, these populations can continue to be exposed through inhalation of re-suspended particles [6–8].

Recent evidence implicates environmental exposure to several individual airborne metals and adverse respiratory outcomes. A recent study found that children living in Guiyu, China, an e-waste processing town with high ambient Mn, Ni and Cr, had significantly lower lung function measures than children living in a town with no history of e-waste processing [9]. In the United States, the risk of cardiovascular and respiratory hospitalizations was found to be higher in counties with higher measured levels of Ni [10]. In New York City, central site ambient measures of Ni were associated with increased probability of wheeze in children aged 24 months [11]. Central site levels of ambient Zn were associated with increases in emergency department (ED) visits and hospitalizations for asthma in a pediatric population in Baltimore [12]. In California, increased ambient levels of Fe and Zn were associated with respiratory hospital admissions among children [13]. A review of the literature on the respiratory effects of metals in ambient PM also reported that most studies showed an increased risk of respiratory morbidity with increasing concentrations of these metals [14].

Brescia, Italy is an industrial province with a long history of iron and ferroalloy production. Over the past century, ferroalloy emissions have increased the environmental levels of Mn, Ni, Cr, Zn and Fe in this province [15]. We had previously recruited adolescents living in a historic, a current and a non-industrial area of Brescia, as part of a cross-sectional study on behavior, cognitive and motor functions [16, 17]. Given the major sources of exposure in this area, it is important to understand how the individual airborne metals may also impact respiratory health. With this purpose, we investigated the association between personal measures of ambient metals and respiratory health in adolescents.

## Methods

### Study population

Children were enrolled from communities with varying Mn levels: Bagnolo Mella which has an active ferroalloy plant and high ambient Mn levels, Valcamonica, which had three ferroalloy plants until 2001 and Garda Lake which has no history of ferroalloy plant activity. These children were recruited to build upon the existing EU-funded PHIME (Public Health Impact of Mixed element Exposure in susceptible populations) study that had already examined children from Valcamonica and Garda Lake (first phase of recruitment). During the second phase of recruitment, participants were identified through the local school district and recruited through school presentations. The recruitment strategy was driven by participant residence, distance from the ferroalloy plant (Bagnolo Mella) or former plant (Valcamonica), or a randomly selected point (Garda Lake), and the measured gradient of ambient Mn from the plant/former plant. Participants were targeted within each study site to maximize the Mn exposure gradient within each recruitment site and to limit the effects of town of residence on the analysis. Also, the same percentage of males and females in each group were obtained through frequency based matching. Participants were enrolled if they met the following inclusion criteria: were born in the respective area to a family who resided in the area for at least a generation, had lived in the study area since birth and were aged between 11 and 14 years. These ages were chosen because the original study was conceived to examine neurocognitive outcomes. This is the age of pre-adolescence, characterized by the onset of anxiety symptoms and gender differences. It also coincides with the years of mid-school. This age group may be exposed to environmental pollutants differently from childhood and adolescence. Exclusion criteria included: known hand or finger motor deficits, visual deficits not adequately corrected and any history of neurological, metabolic, hepatic or endocrine diseases. Children were also excluded if they had a history of receiving parenteral nutrition that may cause Mn overload, were currently taking prescription psychoactive drugs or had known psychiatric disturbances. Informed consent was obtained from parents and children. Study protocols were approved by the institutional review boards at the Ethical Committee of the Public Health Agency of Brescia, University of Brescia, University of California-Santa Cruz, and Harvard School of Public Health.

### Exposure assessment

PM_10_ was collected continuously on commercially available filters (37 mm diameter, PTFE-Teflon) for 24 h using Personal Environmental Monitors (SKC Inc., 84, PA) carried in a small backpack by each participant. The PEM air sampler was attached to a participant’s backpack strap, located near the breathing zone and connected to a Leland Legacy pump inside the backpack with a pre-set flow rate of 10 l/min (SKC Leland Legacy).

The participants carried the backpack or placed it nearby while in school or sleeping. Each participant was also instructed to record their activities and locations while carrying the monitor.

Metals in filters were measured using a non-destructive method described previously [18]. Briefly, each filter sample was placed between two thin polypropylene sheets and measurements were performed using a Bruker TXRF system S2 Picofox (Billerica, MA). Absolute elemental concentrations were evaluated using an external air particulate standard filter from the National Institute of Standards and Technology of the U.S. (NIST-SRM 2783).

### Questionnaires

Questionnaires about respiratory illness were distributed to the parents of the participants only during the second phase of recruitment. The questionnaire used was originally developed for the Viadana study (http://biometria.univr.it/viadanastudy/questionari/EN_viadquest.pdf), a cross-sectional survey established to investigate the associations between proximity to chipboard and wood manufacturing industries and several health outcomes, including respiratory symptoms in children living in Viadana, Italy [19]. Questions were compiled from previously validated questionnaires with available Italian translations, which included the International Study for Asthma and Allergies in Childhood (ISAAC, 13–14 year old age group), European Community Respiratory Health Survey (ECRHS), Studi Italiani sui Disordini Respiratori dell’Infanzia e l’Ambiente (SIDRIA [20]) and indoor climate MM questionnaires (MM040NA and MM080, [21]). Questionnaires on demographic data were also filled out by the parents of the participants.

### Statistical analyses

Data were analyzed using a modified Poisson regression with a robust error estimator [22]. The outcomes of interest were parental report of asthma, report of asthma medication use in the last 12 months, report of wheeze in the past 12 months, and report of nasal allergies/hay fever in the last 12 months. For the report of asthma outcome, children whose last reported asthma attack was before age 6 were excluded from analysis. This was done in order to exclude children with transient wheeze that resolves early in childhood and is not defined as asthma [23]. Metals were treated as continuous predictors for each of the outcomes. Socioeconomic status was derived from parents’ education and occupation data based on a previously published approach [24]. Briefly, education was divided into three levels: low (elementary and junior high school), medium (senior high school) and high (degree and post-degree). Occupations were grouped into three categories, according to a hypothetical ordinal scale, that considered criteria of the International Classification, socio-economic situation of Italy (and in particular of Lombardy Region) and variables like decision latitude and job demand. Criteria followed indications from the Italian National Institute for Statistics (ISTAT) and an agreement between five independent researchers. The combination of education and occupation levels was used to obtain three levels of the socio-economic index: low, medium and high. To obtain the final SES index the higher level of education and occupation between mother and father were combined. Children whose mother or father was unemployed or deceased were classified in the low SES category. Season of personal monitoring, day of the week and maternal smoking habits (former/never smoker vs. current smoker) and report of eczema symptoms in the past 12 months were also considered as potential covariates but their inclusion in the model did not change the magnitude of the associations between the metals and the selected outcomes and they were not included in the final model. Final multivariable models included the following covariates: report of maternal asthma, child’s sex, child’s age, SES status and in two-pollutant models, PM_10_ concentration. In sensitivity analysis, mixed models with random effects for site were also tested but are not presented because they yielded similar conclusions and based on information criteria did not provide a better model fit. Data were analyzed using SPSS 22 (Chicago, IL) and R statistical package. Risk ratios for outcomes of interest were calculated for an IQR increase in each of the pollutants of interest.

## Results

### Characteristics of cohort and pollutant concentrations

A total of 410 participants were enrolled during the second phase of recruitment. 373/410 (91 %) returned the respiratory questionnaire and 280/373 (75 %) had corresponding personal environmental monitoring data and complete covariate data. There were no significant differences between included and excluded participants from analyses by sex, age, maternal asthma, SES, study site or any of the outcomes of interests (Additional file 1: Table S1). Descriptive characteristics for the participants stratified by study site can be seen in Table 1. There were no significant differences across study sites except that a slightly higher percentage of children lived in an urban area in Bagnolo Mella and Valcamonica when compared to Garda Lake. Overall, 10/269 (4 %) had report of wheezing in the last 12 months, 18/280 (6 %) had a report of asthma medication use in the last 12 months, 35/280 (13 %) children had a report of asthma and 38/277 (14 %) had report of nasal allergies/hay fever in past 12 months.

**Table 1:** Comparison of population characteristics by site

	Bagnolo Mella	Valcamonica	Garda Lake	*p*-value
	*n* = 145	*n* = 80	*n* = 55	
Male sex^a^, *n* (%)	71 (49)	43 (54)	34 (62)	0.262
Age^b^, year, median [25–75^th^]	12 [11–13]	12 [11–13]	12 [12–13]	0.978
SES index^a^, *n* (%)				0.466
Low	29 (20)	19 (24)	6 (11)	
Medium	79 (55)	42 (53)	34 (62)	
High	37 (25)	19 (24)	15 (27)	
Maternal asthma^a^, *n* (%)	15 (10)	6 (8)	5 (9)	0.779
Residence in urban area^a^, n (%)	130 (95)	64 (89)	40 (83)	0.040
Mother smokes^a^, n (%)	33 (23)	20 (25)	9 (16)	0.457
PM_10_^b^ (μg/m^3^), median [25–75^th^]	63.1 [48.5–88.8]	65.7 [51.8–88.9]	52.1 [37.6–71.3]	0.004
Mn^b^ (ng/m^3^), median [25–75^th^]	33.4 [19.7–91.8]	22.9 [10.9–44.3]	14.7 [7.61–28.9]	0.000
Ni^b^ (ng/m^3^), median [25–75^th^]	3.70 [2.10–6.66]	3.36 [1.67–6.90]	1.97 [1.15–3.84]	0.001
Cr^b^ (ng/m^3^), median [25–75^th^]	8.06 [4.86–14.3]	5.56 [3.29–10.7]	7.25 [3.66–11.0]	0.030
Fe^b^ (ng/m^3^) median [25–75^th^]	346 [220–629]	559 [278–1058]	305 [173–479]	0.002
Zn^b^ (ng/m^3^), median [25–75^th^]	80.9 [47.0–118]	56.1 [27.7–107]	57.6 [36.0–92.9]	0.061

^a^ Differences tested using Pearson Chi-Square test

^b^ Differences tested using Kruskal-Wallis test

**Fig. 1:**
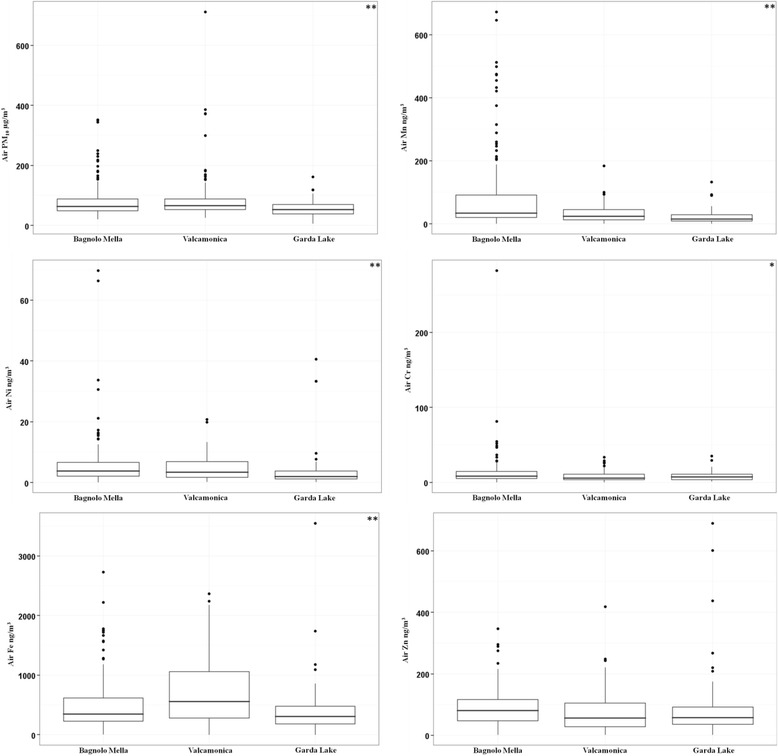
Box plots of pollutant concentrations by study site. Kruskal Wallis Test. *p <0.05. **p <0.01

Pollutant concentrations stratified by site are described in both Fig. 1 and Table 1. Concentrations of Mn, Ni, Fe, Cr and PM_10_ were significantly different by site (Kruskal-Wallis Test *p*-value <0.05 for all). Zn did not significantly vary by site. Concentrations of Mn, Ni, Cr and Zn were highest Bagnolo Mella. Fe concentrations were highest in Valcamonica as a result of emissions from historical alloy production that always includes Fe. Although not directly comparable, the occupational exposure limits for Mn, Ni and Cr are presented in Additional file 2. Table 2 shows Spearman correlation values for all pollutants. All metals were highly and significantly correlated to one another (correlation values all >0.7). PM_10_ was only moderately to weakly correlated with metal concentrations (correlation values between 0.14 and 0.3).

**Table 2:** Spearman correlation matrix for pollutants

	PM_10_	Mn	Ni	Cr	Fe	Zn
PM_10_	1.000	0.236^**^	0.213^**^	0.141^*^	0.296^**^	0.175^**^
Mn		1.000	0.754^**^	0.740^**^	0.721^**^	0.747^**^
Ni			1.000	0.791^**^	0.765^**^	0.775^**^
Cr				1.000	0.717^**^	0.738^**^
Fe					1.000	0.784^**^
Zn						1.000

*p <0.05

**p <0.01

### Association between metals and respiratory outcomes

Table 3 shows the risk ratios for parental report of asthma, report of asthma medication use in last 12 months, report of wheeze in the last 12 months, and nasal allergies/hay fever in last 12 months for an IQR increase in each pollutant. We saw no significant associations between Fe and Zn and any of these respiratory outcomes. We found small significant associations between Mn concentrations (RR: 1.09, 95 % CI [1.09, 1.34] per 42 ng/m^3^ increase) Ni concentrations (RR: 1.11, 95 % CI [1.02, 1.20] per 9 ng/m^3^ increase) and Cr concentrations (RR: 1.08, 95 % CI [1.06, 1.11] and report of asthma. Increased Mn (RR: 1.13, 95 % CI [1.00, 1.24] was significantly associated with report of asthma medication use in the last 12 months. A similar association was seen for Ni concentrations and report of asthma medication use but the results were of borderline statistical significance (*p* = 0.052). PM_10_ concentrations were also independently associated with report of asthma, report of asthma medication use in the past 12 months and report of nasal allergies/hay fever in the past 12 months. There were no significant associations between any of the pollutants and report of wheeze in the past 12 months.

**Table 3:** Single pollutant models for associations between pollutants and respiratory outcomes

Pollutant (IQR)	Report of asthma	Asthma medication use in past 12 months	Wheeze in past 12 months	Nasal allergies/hay fever in past 12 months
PM_10_ (38 μg/m^3^)	1.12* (1.00, 1.21)	1.21** (1.09, 1.35)	0.58 (0.28,1.21)	1.08* (1.00, 1.16)
Mn (42 ng/m^3^)	1.09* (1.00,1.18)	1.13* (1.00, 1.23)	1.09 (0.92, 1.29)	0.96 (0.85, 1.09)
Ni (4 ng/m^3^)	1.11* (1.02, 1.20)	1.11 (1.00, 1.24)	1.00 (0.83, 1.21)	1.00 (0.86, 1.15)
Fe (498 ng/m^3^)	1.00 (1.00, 1.00)	1.00 (1.00, 1.00)	1.00 (0.99, 1.00)	1.00 (1.00, 1.00)
Cr (9 ng/m^3^)	1.08** (1.06, 1.11)	1.06 (0.97, 1.15)	1.06 (0.96, 1.17)	1.03 (0.94, 1.12)
Zn (72 ng/m^3^)	1.00 (0.81, 1.33)	1.15 (0.93, 1.54)	0.75 (0.36, 1.65)	0.93 (0.70, 1.24)

Models adjusted for maternal asthma, child’s sex, child’s age and SES status

*p <0.05

**p <0.01

Given that PM_10_ was only moderately correlated with metal concentrations, models were run with the inclusion of PM_10_ as a co-pollutant. As shown in Table 4, the associations between Mn, Ni, Cr and report of asthma and Mn and report of asthma medication use were virtually unchanged and remained significant after adjustment for concurrent PM_10_ concentrations. The association between Ni and report of asthma medication use achieved statistical significance with the inclusion of PM_10_ in the model.

**Table 4:** Two-pollutant models for associations between pollutants and respiratory outcomes

Pollutant (IQR)	Report of asthma	Asthma medication use in past 12 months	Wheeze in past 12 months	Nasal allergies/hay fever in past 12 months
Mn (42 ng/m^3^)	1.09* (1.00,1.18)	1.13* (1.04,1.29)	1.09 (0.92, 1.29)	0.96 (0.85, 1.09)
Ni (4 ng/m^3^)	1.11** (1.03, 1.21)	1.13* (1.01, 1.27)	1.00 (0.85, 1.17)	1.00 (0.86, 1.17)
Fe (498 ng/m^3^)	1.00 (1.00,1.00)	1.00 (1.00, 1.00)	1.00 (0.99, 1.00)	1.00 (1.00, 1.00)
Cr (9 ng/m^3^)	1.08** (1.06, 1.11)	1.06 (0.97, 1.15)	1.07 (0.97, 1.17)	1.03 (0.94, 1.12)
Zn (72 ng/m^3^)	1.00 (0.81, 1.33)	1.15 (0.87, 1.54)	0.81 (0.39, 1.65)	0.93 (0.70, 1.24)

Models adjusted for maternal asthma, child’s sex, child’s age, SES status and PM_10_ concentration

*p <0.05

**p <0.01

## Discussion

The objective of this study was to characterize the association between personal measures of Mn, Ni, Cr, Zn and Fe and respiratory outcomes in a cross-section of children living in an industrial province in Italy. In this study we found that increasing personal concentrations of Mn, Ni and Cr were associated with increased risk of adverse respiratory outcomes in Italian adolescents. Furthermore, these associations were independent of PM_10_ concentrations and the observed associations remained significant after adjustment for a number of important confounders. These findings add to the growing literature on the effects of PM composition on respiratory health.

Recent studies in Europe have reported on the effects of metals on children’s respiratory health. In a birth cohort in the Netherlands, metal fractions of PM_2.5_ and PM_10_ were measured in filters by x-ray fluorescence and used for development of LUR models for these metals [25]. Concentration of K and S in PM_2.5_ and K and Zn in PM_10_ at the children’s residence were associated with higher risk of incident asthma and these associations appeared to be independent from PM mass [25]. This same study reported associations between annual PM_2.5_ abs, Cu and Fe in PM_2.5_ at children’s current addresses and small but significant decreases in FEV_1_ ranging between 0.9 and 1.1 % [25]. Elemental fractions of PM_10_ and PM_2.5_ were also examined as predictors of lung function measures at ages 6–8 in a meta-analysis of European birth cohorts [26]. In meta- analysis results, there were small reductions in FEV_1_ associated with Ni concentrations in PM_10_ and the associations were independent of PM mass [26]. These studies are in line with a recent review of literature on the respiratory effects of metals in which the epidemiological studies reviewed reported positive associations between increasing metal concentrations, particularly Fe, Ni and Zn, and increased risk of respiratory morbidity [14].

Even though several studies have documented the long-term [27] and acute effects [28, 29] of Mn and Cr from welding fumes on respiratory health, few have focused on the effect of these metals on children’s health. One study in China found that children in an e-waste province in China exposed to Mn, Ni and Cr fumes from an e-waste facility had decreased lung function measures when compared to a reference area, although the effect was only limited to boys in the 8–9 year age group [9]. The authors also reported a significant association between increased blood Mn and serum Ni and decreased forced vital capacity [9]. Another study in South Korea found associations between central site measures of Mn in PM and decreased peak flow measures in school children [30].

One of the mechanisms through which inhaled metals may lead to airway disease is through oxidative stress. A study in Germany reported increased secretion of pro-inflammatory cytokines interleukin (IL)-6 and tumor necrosis factor (TNF)-α and elevated levels of oxidant radicals in bronchoalveolar lavage fluid (BAL) after in vivo instillation of ambient particles rich in Ni, Zn, Cd and Cu [31]. The e-waste study in China also found significant associations between increased blood Mn and serum Ni and increased malondialdehyde, a marker of lipid peroxidation [9]. Another potential mechanism by which these metals may affect the respiratory system involves the dysregulation of Fe homeostasis in the lungs. Inhaled Ni may compete with the uptake of endogenous Fe, leading to elevated levels of available Fe and release of reactive oxygen species (ROS) [32, 33]. Recent exposure to ambient metals has also been associated with increases in airway inflammation in inner-city children living in NYC [34]. Ni concentrations in particular were associated with increases in distal airway inflammation and such inflammation has been be associated airway hyper-responsiveness and symptom exacerbation [35, 36].

We did not see any associations between Fe and Zn and any respiratory outcome. A potential explanation for the lack of effect is the distribution of these metals on different-sized particles. In a study of airborne pollutants in Greece, Ni and Mn were found in fine, intermediate and coarse airborne particles, with Ni being predominant in the alveolar fraction of PM (<0.8 μm), while Fe was mainly found in particles with a diameter >2.7 μm [37]. These findings suggest that Fe might not travel as deep into the airways as Mn and Ni. Another potential explanation is that we did not see significant differences in Zn concentrations across the 3 study sites. While Fe and Zn were the most abundant metals in PM_10_, Mn, Ni and Cr concentrations were much higher than concentrations reported in other epidemiological studies [14]. We also did not see any associations with report of wheeze in the past 12 months; perhaps due to the low prevalence of this outcome in our cohort we did not have sufficient statistical power to detect any associations.

Even though we have focused on the role of ferroalloy emissions as the source for these ambient metals, these metals are also emitted during a variety of other processes. Motor vehicle emissions are important sources of ambient Mn, Fe, Cr and Zn [38, 39]. Other sources of ambient Fe include oil combustion, road dust, and re-suspended soils [38, 40]. Ni is also released during oil combustion and other industrial activities [41]. However in support of ferroalloy emissions as the major source in our study area, we previously reported on fingerprint analyses used to identify exposure sources as potentially related to industrial vs. traffic emission, based on specific interrelations of elements [42]. Principal Component Analysis confirmed the industrial origin of these metal emissions [42].

There are several strengths to our study. We were able to examine the effect of several metals on respiratory health. The associations between metals and respiratory outcomes remained significant after adjustment for PM_10_ concentrations. Ambient air pollution studies have typically relied on central-site monitoring data or modeling exposure variables. The use of personal monitoring may provide a better representation of actual daily exposure; a study in NYC schoolchildren found the strongest associations to be between personal measures of elemental carbon (EC) and asthma symptoms, when compared to EC exposure measures from stationary school monitors [43].

There are also some limitations to our study. Our sample size was relatively small and personal sampling was only done for 24 h and we were not able to control for long-term exposure to these pollutants. However, children are likely to spend most of their time in their neighborhood (typically at home and school) and they travel less than adults. Moreover, adjusting for season and day of the week did not change our association estimates. We cannot rule out that ambient metals may be surrogates for other components found in PM_10_ or other pollutants that are known to be correlated with PM (*ie* NO_2_). The effect sizes for these associations were small and our analyses were also cross-sectional in nature, and we cannot prove causality. The study outcomes were not objectively assessed but reported by the parents. Thus, it could be argued that health risk perception in the population may be a source of bias [44]. However, when further adjusting the analyses for an indicator of health risk perception [44] our conclusions were unchanged. Asthma questionnaires have a low sensitivity, however their high specificity ensures that the children who report the outcomes were correctly identified [45]. Our results are also in line with published data from both SIDRIA 1 and 2 (ISAAC Italy) where the prevalence of self/parent reported asthma in the 13–14 year old population was around 10 % [46, 47]. It is also important to highlight that the association estimates of Ni and Mn concentrations were similar when analyzing a lifetime asthma vs current asthma. Finally, our population was Caucasian and the majority were of medium-high SES, limiting the generalizability of our results.

## Conclusions

In conclusion, the associations between 24-hour personal measures of airborne Mn, Ni and Cr and report of wheeze and doctor diagnosed asthma suggest that these metal fractions may affect respiratory health. These results highlight the importance of analyzing the composition of inhalable particulate matter in order to better understand what specific components may be responsible for detrimental health effects. Knowledge of the effects of these different metals may aid in the development of more targeted interventions directed at their sources.

## Additional files

Additional file 1: Table S1. Selected cohort characteristics. Comparison of baseline characteristics between included and excluded study participants. (DOCX 13 kb) Additional file 2: Table S2. TWA recommendations from the European Commission of Employment, Social Affairs and Inclusion’s Scientific Committee on Occupational Exposure Limits (SCOEL). (DOCX 11 kb)

## Abbreviations

ISAAC: International Study for Asthma and Allergies in Childhood
PHIME: Public Health Impact of Mixed element Exposure in susceptible populations
PM_10_: Particulate matter <10 microns in diameter
SES: Socioeconomic Status

## References

[1] Haluza D, Moshammer H, Hochgatterer K (2014). Dust is in the air. Part II: effects of occupational exposure to welding fumes on lung function in a 9-year study. Lung.

[2] Wittczak T, Dudek W, Walusiak-Skorupa J, Swierczynska-Machura D, Cader W, Kowalczyk M (2012). Metal-induced asthma and chest X-ray changes in welders. Int Arch Occup Environ Health.

[3] Jafari AJ, Assari MJ (2004). Respiratory effects from work-related exposure to welding fumes in Hamadan, Iran. Arch Environ Health.

[4] Hedmer M, Karlsson JE, Andersson U, Jacobsson H, Nielsen J, Tinnerberg H (2014). Exposure to respirable dust and manganese and prevalence of airways symptoms, among Swedish mild steel welders in the manufacturing industry. Int Arch Occup Environ Health.

[5] Dales R, Kauri LM, Cakmak S, Mahmud M, Weichenthal SA, Van Ryswyk K (2013). Acute changes in lung function associated with proximity to a steel plant: A randomized study. Environ Int.

[6] Young TM, Heeraman DA, Sirin G, Ashbaugh LL (2002). Resuspension of soil as a source of airborne lead near industrial facilities and highways. Environ Sci Technol.

[7] Harris AR, Davidson CI (2005). The role of resuspended soil in lead flows in the California South Coast Air Basin. Environ Sci Technol.

[8] Pavilonis BT, Lioy PJ, Guazzetti S, Bostick BC, Donna F, Peli M (2015). Manganese concentrations in soil and settled dust in an area with historic ferroalloy production. J Expo Sci Environ Epidemiol.

[9] Zheng GN, Xu XJ, Li B, Wu KS, Yekeen TA, Huo X (2013). Association between lung function in school children and exposure to three transition metals from an e-waste recycling area. J Expo Sci Env Epid.

[10] Bell ML, Ebisu K, Peng RD, Samet JM, Dominici F (2009). Hospital admissions and chemical composition of fine particle air pollution. Am J Respir Crit Care Med.

[11] Patel MM, Hoepner L, Garfinkel R, Chillrud S, Reyes A, Quinn JW (2009). Ambient metals, elemental carbon, and wheeze and cough in New York City children through 24 months of age. Am J Respir Crit Care Med.

[12] Hirshon JM, Shardell M, Alles S, Powell JL, Squibb K, Ondov J (2008). Elevated ambient air zinc increases pediatric asthma morbidity. Environ Health Perspect.

[13] Ostro B, Roth L, Malig B, Marty M (2009). The effects of fine particle components on respiratory hospital admissions in children. Environ Health Perspect.

[14] Gray DL, Wallace LA, Brinkman MC, Buehler SS, La Londe C (2015). Respiratory and cardiovascular effects of metals in ambient particulate matter: a critical review. Rev Environ Contam T.

[15] Zacco A, Resola S, Lucchini R, Albini E, Zimmerman N, Guazzetti S (2009). Analysis of settled dust with X-ray Fluorescence for exposure assessment of metals in the province of Brescia, Italy. J Environ Monitor.

[16] Lucchini RG, Guazzetti S, Zoni S, Donna F, Peter S, Zacco A (2012). Tremor, olfactory and motor changes in Italian adolescents exposed to historical ferro-manganese emission. Neurotoxicology.

[17] Lucchini RG, Zoni S, Guazzetti S, Bontempi E, Micheletti S, Broberg K (2012). Inverse association of intellectual function with very low blood lead but not with manganese exposure in Italian adolescents. Environ Res.

[18] Borgese L, Zacco A, Pal S, Bontempi E, Lucchini R, Zimmerman N (2011). A new non-destructive method for chemical analysis of particulate matter filters: The case of manganese air pollution in Vallecamonica (Italy). Talanta.

[19] de Marco R, Marcon A, Rava M, Cazzoletti L, Pironi V, Silocchi C (2010). Proximity to chipboard industries increases the risk of respiratory and irritation symptoms in children The Viadana study. Sci Total Environ.

[20] Simoni M, Lombardi E, Berti G, Rusconi F, La Grutta S, Piffer S (2005). Mould/dampness exposure at home is associated with respiratory disorders in Italian children and adolescents: the SIDRIA-2 Study. Occup Environ Med.

[21] Andersson K. Epidemiological approach to indoor air problems. Indoor Air. 1998;32–39.

[22] Zou G (2004). A modified poisson regression approach to prospective studies with binary data. Am J Epidemiol.

[23] Martinez FD (2002). Development of wheezing disorders and asthma in preschool children. Pediatrics.

[24] Cesana GC, Ferrario M, De Vito G, Sega R, Grieco A (1995). [Evaluation of the socioeconomic status in epidemiological surveys: hypotheses of research in the Brianza area MONICA project]. Med Lav.

[25] Gehring U, Beelen R, Eeftens M, Hoek G, de Hoogh K, de Jongste JC (2015). Particulate matter composition and respiratory health: the PIAMA Birth Cohort Study. Epidemiology.

[26] Eeftens M, Hoek G, Gruzieva O, Mölter A, Agius R, Beelen R (2014). Elemental composition of particulate matter and the association with lung function. Epidemiology.

[27] Boojar MMA, Goodarzi F (2002). A longitudinal follow-up of pulmonary function and respiratory symptoms in workers exposed to manganese. J Occup Environ Med.

[28] Sobaszek A, Boulenguez C, Frimat P, Robin H, Haguenoer JM, Edme JL (2000). Acute respiratory effects of exposure to stainless steel and mild steel welding fumes. J Occup Environ Med.

[29] Walters GI, Moore VC, Robertson AS, Burge CBSG, Vellore AD, Burge PS (2012). An outbreak of occupational asthma due to chromium and cobalt. Occup Med-Oxford.

[30] Hong YC, Hwang SS, Kim JH, Lee KH, Lee HJ, Lee KH (2007). Metals in particulate pollutants affect peak expiratory flow of schoolchildren. Environ Health Perspect.

[31] Schaumann F (2004). Metal-rich ambient particles (Particulate Matter2.5) cause airway inflammation in healthy subjects. Am J Respir Crit Care Med.

[32] Ghio AJ, Cohen MD (2005). Disruption of iron homeostasis as a mechanism of biologic effect by ambient air pollution particles. Inhal Toxicol.

[33] Prophete C, Maciejczyk P, Salnikow K, Gould T, Larson T, Koenig J (2006). Effects of select PM-associated metals on alveolar macrophage phosphorylated ERK1 and −2 and iNOS expression during ongoing alteration in iron homeostasis. J Toxicol Environ Health A.

[34] Rosa MJ, Perzanowski MS, Divjan A, Chillrud SN, Hoepner L, Zhang HJ (2014). Association of recent exposure to ambient metals on fractional exhaled nitric oxide in 9–11 year old inner-city children. Nitric Oxide-Biol Ch.

[35] Martin RJ (2002). Therapeutic significance of distal airway inflammation in asthma. J Allergy Clin Immunol.

[36] Kraft M, Pak J, Martin RJ, Kaminsky D, Irvin CG (2001). Distal lung dysfunction at night in nocturnal asthma. Am J Resp Crit Care.

[37] Samara C, Voutsa D (2005). Size distribution of airborne particulate matter and associated heavy metals in the roadside environment. Chemosphere.

[38] Li Z, Hopke PK, Husain L, Qureshi S, Dutkiewicz VA, Schwab JJ (2004). Sources of fine particle composition in New York city. Atmos Environ.

[39] Poulakis E, Theodosi C, Bressi M, Sciare J, Ghersi V, Mihalopoulos N (2015). Airborne mineral components and trace metals in Paris region: spatial and temporal variability. Environ Sci Pollut Res Int.

[40] Chillrud SN, Epstein D, Ross JM, Sax SN, Pederson D, Spengler JD (2004). Elevated airborne exposures of teenagers to manganese, chromium, and iron from steel dust and New York City’s subway system. Environ Sci Technol.

[41] Hertel RF, Maas T, Muller VR. Environmental Health Criteria 108: Nickel. World Health Organization; 1991.

[42] Borgese L, Federici S, Zacco A, Gianoncelli A, Rizzo L, Smith DR (2013). Metal fractionation in soils and assessment of environmental contamination in Vallecamonica, Italy. Environ Sci Pollut R.

[43] Spira-Cohen A, Chen LC, Kendall M, Lall R, Thurston GD (2011). Personal exposures to traffic-related air pollution and acute respiratory health among Bronx schoolchildren with asthma. Environ Health Perspect.

[44] Marcon A, Nguyen G, Rava M, Braggion M, Grassi M, Zanolin ME (2015). A score for measuring health risk perception in environmental surveys. Sci Total Environ.

[45] Yang CL, To T, Foty RG, Stieb DM, Dell SD (2011). Verifying a questionnaire diagnosis of asthma in children using health claims data. BMC Pulm Med.

[46] Renzoni E, Forastiere F, Biggeri A, Viegi G, Bisanti L, Chellini E (1999). Differences in parental- and self-report of asthma, rhinitis and eczema among Italian adolescents. Eur Respir J.

[47] Migliore E, Pearce N, Bugiani M, Galletti G, Biggeri A, Bisanti L (2007). Prevalence of respiratory symptoms in migrant children to Italy: the results of SIDRIA-2 study. Allergy.

