# Supplementary material for: Association between personal exposure to ambient metals and respiratory disease in Italian adolescents: a cross-sectional study
Source: BMC Pulm Med. 2016 Jan 12;16:6. doi: 10.1186/s12890-016-0173-9 (PMC4709999; doi:10.1186/s12890-016-0173-9)
Supplement: Additional file 1: Table S1. — Selected cohort characteristics. Comparison of baseline characteristics between included and excluded study participants. (DOCX 13 kb) [file 12890_2016_173_MOESM1_ESM.docx]

**Table S1.** Selected cohort characteristics

|  | Included  n=280 | Excluded  n=130 | p-value^a^ |
| --- | --- | --- | --- |
| Male sex, *n* (%) | 148 (53) | 53 (50) | 0.558 |
| Age, median (25^th^-75^th^) | 12 (12-13) | 12 (12-13) | 0.455 |
| SES index, *n* (%)  Low  Medium  High | 54 (19)  155 (55)  71 (25) | 26 (29)  45 (50)  19 (21) | 0.152 |
| Site,  Bagnolo Mella  Valcamonica  Garda Lake | 145 (52)  80 (29)  55 (20) | 69 (54)  25 (20)  34 (27) | 0.091 |
| Maternal asthma, *n* (%) | 26 (9) | 10 (11) | 0.678 |
| Child ever wheezed, *n* (%) | 64 (24) | 29 (28) | 0.384 |
| Wheeze in last 12 months, *n* (%) | 10 (4) | 3 (3) | 0.705 |
| Doctor said child had asthma, *n* (%) | 35 (13) | 9 (10) | 0.428 |
| Nasal allergies/hay fever in last 12 months, *n* (%) | 38 (14) | 10 (10) | 0.310 |

^a^ Differences in categorical variables tested using Pearson Chi-Square, differences in continuous variables tested using Mann Whitney U test

In the excluded stratum, 23/130 (18%) participants were missing sex and age data, 37/130 (29%) were missing maternal asthma, 40/130 (31%) were missing SES status, 2/130 (2%) missing site, 27/130 (21%) were missing ever wheeze, current wheeze, eczema, nasal allergies data and 35/130 (27%) were missing doctor said child has asthma.
