# Supplementary material for: Association between personal exposure to ambient metals and respiratory disease in Italian adolescents: a cross-sectional study
Source: BMC Pulm Med. 2016 Jan 12;16:6. doi: 10.1186/s12890-016-0173-9 (PMC4709999; doi:10.1186/s12890-016-0173-9)
Supplement: Additional file 2: Table S2. — TWA recommendations from the European Commission of Employment, Social Affairs and Inclusion’s Scientific Committee on Occupational Exposure Limits (SCOEL). (DOCX 11 kb) [file 12890_2016_173_MOESM2_ESM.docx]

**Table S2.** TWA* recommendations from the European Commission of Employment, Social Affairs and Inclusion’s Scientific Committee on Occupational Exposure Limits (SCOEL)

| **Pollutant** | **Respirable fraction (<10 microns )** |
| --- | --- |
| Mn | 0.05 mg/m^3^ |
| Ni | 0.01 mg Ni/m^3^ |
| Cr | 2.0 mg/m^3^ total dust (calculated as Cr) |

*The time-weighted average (TWA) exposure limit - the maximum average concentration of a chemical in air for a normal 8-hour working day and 40-hour week
